# Icaritin inhibits osteoclast differentiation and reduces bone loss by targeting ESR1 to inhibit miR503/RANK pathway

**DOI:** 10.3389/fphar.2025.1603333

**Published:** 2025-09-12

**Authors:** Baoping Xie, Xiaofei Liao, Liuyan Xin, Zhen Xie, Qi Jin, An Li, Hongliang Li, Jinping Li

**Affiliations:** ^1^ Key Laboratory of Prevention and Treatment of Cardiovascular and Cerebrovascular Diseases of Ministry of Education, Jiangxi Provincial Key Laboratory of Tissue Engineering, Gannan Medical University, Ganzhou, Jiangxi, China; ^2^ Department of Pharmacy, Ganzhou People’s Hospital, Ganzhou, Jiangxi, China; ^3^ Department of Oncology, First Affiliated Hospital of Gannan Medical University, Ganzhou, Jiangxi, China; ^4^ Department of Pharmachemistry, Xiangya School of Pharmaceutical Sciences, Central South University, Changsha, Hunan, China

**Keywords:** icaritin, osteoclast, MiR-503-5p, estrogen receptor, osteoporosis

## Abstract

**Background:**

Postmenopausal osteoporosis (PMOP) is a prevalent metabolic disorder characterized by pathogenic mechanisms associated with the dysfunction of osteoclasts (OC) and osteoblasts (OB). Icaritin (ICT) is a flavonoid derived from icariin and epimedium, which is a natural product, and has demonstrated promising anti-osteoporosis properties. Nevertheless, the targets and mechanisms of ICT in osteoclast differentiation and PMOP remain unclear.

**Methods:**

we developed a bilateral ovariectomy-induced osteoporosis model in animals and receptor activator of nuclear factor kappa-B ligand (RANKL) induced RAW264.7 to differentiate into osteoclasts with or without MPP dihydrochloride (MPP) and antagomir-503-5p. Micro-CT, tartrate-resistant acid phosphatase (TRAP) staining, enzyme-linked immunosorbent assay (ELISA), Western blot and qRT-PCR were used to detect bone resorption function, bone metabolism parameters, osteoclast differentiation rate and the expression of related genes, as well as the expression of ESR1, miR-503 and RANK. Molecular docking, cell thermal shift assay (CETSA) and drug affinity responsive target stability (DARTs) experiments were used to confirmed that ESR1 is the direct target of ICT, and binding site of ICT with ESR1.

**Results:**

ICT significantly inhibited OC differentiation and the expression of related genes (*Trap*, *Mmp9*, and *Nfatc1*), reduced bone loss, and improved osteoporosis and bone trabecular structure, and inhibited the levels of TRAP and RANKL in the serum and increase the level of osteoprotegerin (OPG). ICT significantly enhanced the expression of ESR1, ESR2 and miR-503, while inhibiting RANK expression, and ESR1 is the direct target of ICT, and Asparagine at 455 is the direct binding site of ICT with ESR1. Moreover, blocking ESR1 significantly reduced the regulatory effect of ICT on OC differentiation and related gens expression by MPP, especially the expression of miR-503 and RANK, as well as weakened the regulatory effect of ICT on inhibiting bone loss. Antagomir-503-5p significantly reduced the regulatory effect of ICT on OC differentiation, as well as the expression of genes related to OC differentiation.

**Conclusion:**

Taken together, our study confirmed that ESR1 is the direct target of ICT, and Asparagine at 455 is the direct binding site of ICT, and ICT inhibits OC differentiation and reduces bone loss by targeting ESR1 to upregulate miR503 level and weaken miR503/RANK pathway.

## Graphical Abstract

**Figure:**
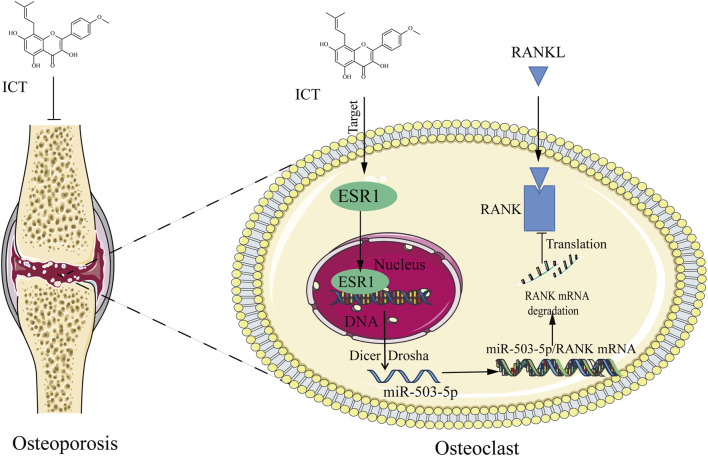
ESR1 is the direct target of ICT, and Asparagine at 455 is the direct binding site of ICT. ICT inhibit osteoclast differentiation and reduce bone loss by targeting ESR1 to upregulate miR-503 level and weaken miR-503/RANK pathway.

## 1 Introduction

The skeletal system is an active tissue that undergoes constant remodeling throughout an individual’s lifespan. Especially osteoporosis (OP) and postmenopausal osteoporosis (PMOP), this process of OP is regulated by the balance between osteoblasts (OB) and osteoclasts (OC) (Andreev et al., 2020). OC are multinucleated giant cell derived from hematopoietic stem cells, which are the only cells with bone absorption function *in vivo*, and induced by macrophage colony-stimulating factor (MCSF) and receptor activator of nuclear factor kappa-B ligand (RANKL) (Boyce, 2013). Consequently, targeting osteoclasts has emerged as a potential therapeutic approach for OP clinical therapy (Xu et al., 2016).

In postmenopausal women, the main cause of PMOP is inadequate estrogen production, which can also be induced in animal models by ovariectomize (OVX) (Geng et al., 2019; Tao et al., 2021a). Moreover, conditional deletion of estrogen receptor α (ESR1) in skeletal muscle leads to osteopenia by increasing the ratio of osteoclast surface to the bone surface. Therefore, the ESR signaling pathway plays a critical role in the treatment of OP. Moreover, accumulating researches have shown that icaritin (ICT) promotes MC3T3-E1 osteogenic differentiation by activating the ERK1/2 and p38 signaling pathways mediated by the ESR (Wu et al., 2017; Norton et al., 2022), and studies confirmed that ICT ameliorates osteoclastogenesis and ovariectomy-induced OP (Huang et al., 2023). These results suggest that ICT has an anti-osteoporotic effect by regulating the balance between OB and OC; the mechanism may be related to the ESR signaling pathway. More interestingly, ESR is a class of transcription factors, which can regulate gene expression, including the regulation of microRNA (miR)-503. The latter is a negative regulator of OC differentiation and its overexpression significantly inhibits OC differentiation (Chen et al., 2014; Wang et al., 2021). Furthermore, miR-503 directly targets receptor activator of nuclear factor κ-Β (RANK, *Tnfrsf11a*), which is the receptor responsible for the induction of RANKL by OC differentiation (Wang et al., 2021). In conclusion, the ESR/miR-503/RANK signaling pathway may represent a novel mechanism for regulating OC differentiation.

Icaritin is a bioactive compound found in the Traditional Chinese Medicine (TCM) epimedium (Gao and Zhang, 2022). *In vitro* and *in vivo* researches have shown that ICT is a metabolite of icariin, which can activate immune cells and inhibit the release of inflammatory factors (Bi et al., 2022). ICT induces antitumor immune responses in hepatocellular carcinoma by downregulating alpha fetoprotein gene expression and exacerbating mitophagy and synergizes with doxorubicin to induce immunogenic cell death in hepatocellular carcinoma (Yu et al., 2020). Recently, emerging research has confirmed that ICT inhibits OC differentiation and bone resorptive activity; it also promotes the osteogenic differentiation and maturation of OB (Liu et al., 2014; Wu et al., 2017). However, the specific mechanism and direct target by which ICT inhibits OC differentiation and PMOP have not been elucidated. Previous studies have found that ICT, as a phytoestrogen, has estrogenomimetic effects (Su et al., 2025; Wang et al., 2013). However, the regulatory mechanism of ICT-targeted ESR, especially the mechanism by which ICT-targeted ESR regulates miR503/RANK pathway to inhibit osteoclast differentiation, has not been elucidated. In the present study, we tested the estrogenomimetic effects of ICT, and confirmed the pharmacological effects of ICT on inhibiting osteoclast differentiation and anti-osteoporosis through *in vivo* and *in vitro*, and the expression of ESR1, ESR2 and miR-503 in OC was assessed. More importantly, we first time confirmed that ESR1 is a direct target of ICT through unmodified small-molecule target confirmation methods, and the ICT-ESR1 binding site through plasmid mutation. Mechanically, the function of ESR1 and miR-503 were inhibited using the competitive inhibitor MPP dihydrochloride (MPP) and antagomiR-503-5p to elucidate the mechanism of ICT inhibits OC differentiation and reduce bone loss by targeting ESR1 via the miR-503/RANK axis.

## 2 Materials and methods

### 2.1 *In vivo* study

#### 2.1.1 Animal feeding

In total, forty-nine female wild-type C57BL/6J mice (25 ± 2 g) between 6 and 8 weeks of age were purchased from JiangSu Huachuang Sino Laboratory Animal company, and maintained in specific pathogen-free conditions in a temperature-controlled (23 °C ± 2 °C) room with a relative humidity of 55 ± 15% on a 12 h light/dark cycle with free access to food and water in independent ventilation cages (IVC). The feed, water and bedding should be changed every 3 days, and all the feed, water and bedding are sterilized through high-temperature and high-pressure treatment. This study^,^s protocols were in strict adherence to ethical standards and national guidelines for animal care and use, approved by the Animal Experimentation Ethics Committee of Gannan Medical University (No. 20250458).

#### 2.1.2 Animal experiment and drug administration

**FIGURE 1:**
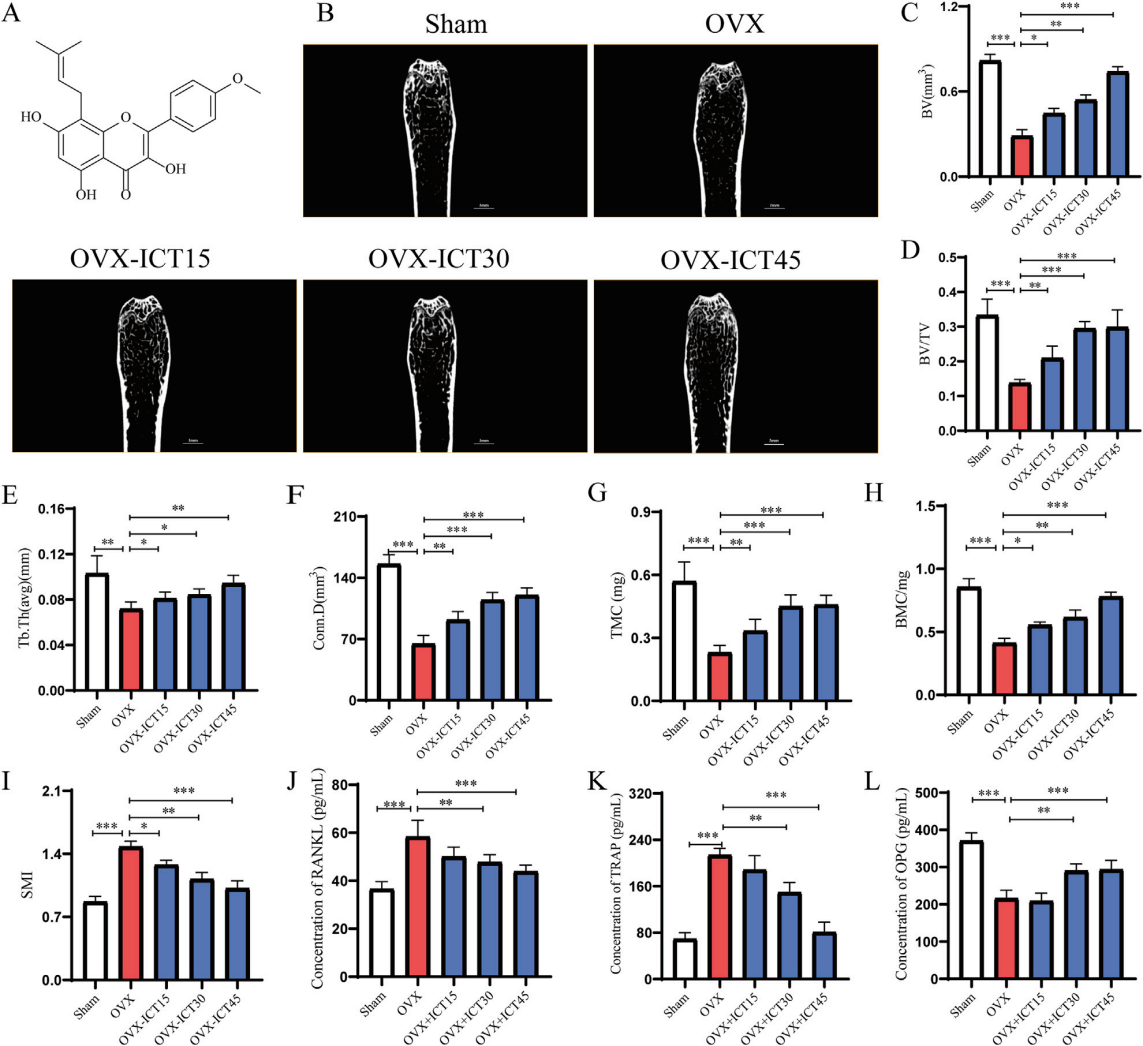
Stereological parameter of trabecular bone in the mouse femur after ICT treatment analyzed by micro-CT. **(A)** Chemical structure of ICT, **(B)** Region of interest (ROI) and longitudinal section, **(C)** Bone volume (BV), **(D)** Bone volume/total volume (BV/TV), **(E)** Trabecular thickness (Tb.Th), **(F)** Trabecular bone connection density (Conn.D), **(G)** Bone mineral content (BMC), **(H)** Tissue mineral content (TMC), **(I)** Structure model index (SMI), **(J–L)** The level of TRAP, RANKL and OPG in serum after ICT treatment. Data are expressed as the mean ± SD (n = 7), *P < 0.05, **P < 0.01, ***P < 0.001 vs. OVX group, scale column: 1 mm.

All animals 1-week adaptation. Then, randomly assigned to seven groups (n = 7/group) for subsequent oral gavage treatments: SHAM-operated (SHAM), OVX-mice (OVX), OVX-mice treated with 15 mg/kg/day ICT (OVX-ICT15), OVX-mice treated with 30 mg/kg/day ICT (OVX-ICT30), OVX-mice treated with 45 mg/kg/day ICT (OVX-ICT45), or OVX-mice treated with 100 μg/kg/day MPP dihydrochloride (OVX-MPP), OVX-mice treated with 15 mg/kg/d ICT and 100 μg/kg/day MPP dihydrochloride (OVX-ICT (Figure 1A) + MPP), while MPP dihydrochloride (HY-103454, ≥98%, MedChemExpress, Shanghai, China) is a selective antagonist of ESR1 and is usually administered by subcutaneous injection (Refaat et al., 2023). Based on our previous research, all animals were anesthetized with isoflurane (RWD LIFE SCIENCE, Shenzhen, China), the surgical instruments were pasteurized, and the surgery was performed in specific pathogen-free conditions, the sham group received no treatment. Fat around ovaries were removed from mice in the Sham group, while bilateral ovaries were removed from mice in other groups (Shi et al., 2024), and the surgical wound should be disinfected with iodophor once a day. All mice were monitored for 8 weeks post-surgery and treatment different concentrations of ICT with or without MPP based on previous studies (Tao et al., 2021b; Hao et al., 2019; Li et al., 2021), and with free access to food and water. After which they were humanely euthanized for the collection and analysis of serum, tibias, and femora. This study^,^s protocols were in strict adherence to ethical standards and national guidelines for animal care and use, approved by the Animal Experimentation Ethics Committee of Gannan Medical University (No. 20250458).

#### 2.1.3 Micro-CT

The left femora of mice were fixed by 4% paraformaldehyde (BL539A, Biosharp, Beijing, China), and bone trabecula in the distal femoral metaphysis was measured by micro-CT [NEMO^®^ Micro-CT, PINGSENG Healthcare (Kunshan) Inc., China]. The scanning targeted the bone trabeculae within the distal femoral metaphysis. The defined scanning area was the distal metaphysis, extending 1 mm proximally from the growth plate, followed by three-dimensional reconstruction. Key indices assessed included bone volume/total volume (BV/TV), Trabecular thickness (Tb.Th), Trabecular bone connection density (Conn.D), Bone mineral content (BMC), Tissue mineral content (TMC), bone volume (BV) and structural model index (SMI). The scanning parameters were set as previous research (Li et al., 2020; Li et al., 2022).

#### 2.1.4 Serum biochemical tests

The blood sample was obtained and centrifugated to separate the serum. The levels of TRAP, RANKL, and OPG in serum were measured using ELISA kit (JINGMEI BIOTECHNOLOGY, Jiangsu, China, Cat: JM-03059M2, JM-02787M2, and JM-02427M2), strictly adhering to the manufacturer’s protocol, and the serum sample can be used directly.

### 2.2 *In vitro* study

#### 2.2.1 Cell culture and toxicity assessment

RAW264.7 and MCF-7 cells were purchased from Zhong Qiao Xin Zhou (Shanghai, China, Cat: ZQ0098 and ZQ0071). RAW264.7 and MCF-7 cells were cultured in high glucose DMEM (Gibco, United States, Cat: C11995500BT), supplemented with 10% fetal bovine serum (FBS; A5669701, Gibco; United States), and 0.1% penicillin (100 U/mL) and streptomycin (100 μg/mL, P1400, Solarbio, China) at 37 °C in the presence of 5% CO_2_, and without RANKL. Toxicity assessment was performed using the Cell Counting Kit-8 (CCK-8; Biosharp, China) reagent to assess the cytotoxicity and estrogen-like effect of icaritin (ICT, A1391, Chengdu Must Biotechnology Co., Ltd., ≥98%, Chengdu, China) and MPP (HY-103454, ≥98%, MedChemExpress, Shanghai, China) on RAW264.7 or MCF-7 cells. Briefly, RAW 264.7 and MCF-7 cells were seeded in 96-well plates (5 × 10^3^ cells per well) for 12 h; all concentrations of ICT (40, 20, 10, 5, 2.5, 1, and 0.1 μM) and MPP (10, 5, 2.5 and 1 μM) were added in 96-well plates and treated for an additional 48 h. The CCK-8 reagent was added to the culture medium, and the absorbance of each group was detected by microplate reader (3020-919, Thermo Fisher Scientific, United States) at a wavelength of 450 nm.

#### 2.2.2 Osteoclastogenesis and tartrate-resistant acid phosphatase (TRAP) staining

To induce OC differentiation *in vitro*, RAW264.7 cells were seeded in 48-well plates (1 × 10^4^ cells per well). The experiment was divided into the control, model and ICT (10 μM, 5 μM and 2.5 μM) groups, with or without ESR1 competitive antagonists MPP (1 μM) and antagomiR-503-5p, according to our previous research (Xie et al., 2019). RAW264.7 cells were cultured in DMEM supplemented with RANKL (50 ng/mL; PeproTech, United States, Cat: 315-11-10 µg). Then, the concentrations of ICT (10 μM, 5 μM and 2.5 μM respectively) were used with or without MPP (1 μM) and antagomiR-503-5p for 5 days. Following RANKL stimulation for 5 days with or without ICT treatment, the cells were washed with PBS buffer 3 times (3 min/times) and fixed in 4% paraformaldehyde (Cat: BL539A, Biosharp, Beijing, China) for 30 min. TRAP staining (Cat. no. PMC-AK04F-COS; Cosmo Bio Co., Ltd, United States) was performed following the instructions of the manufacturer and previous research (Huang et al., 2024), and staining was conducted for 60 min at 37 °C. The TRAP-positive cells exhibited a wine red appearance and ≥3 nuclei were considered for OC counting under the inverted microscope (NIS-Elements F 4.0, United States).

#### 2.2.3 Antagomir-503-5p transfection

RAW264.7 cells were seeded in 6-well plates (4 × 10^4^ cells per well). When the cell growth reached 40% and the cells were adherent for 24 h, antagomir-503-5p (200 nM) or antagomir negative control (200 nM) were transfected into the cells by Lipofectamine 3000 (Cat: L3000015, Invitrogen; United States) and according to the instructions of the kit manufacturer. Subsequently, RAW264.7 cells were treated with ICT (10 μM and 5 μM), RANKL (50 ng/mL) and the culture media were altered every 2 days. Following stimulation of the cells with RANKL for 5 days, according to previous research (Ding et al., 2022). Then, they were used for ACP5 staining or detection of mRNA and protein levels by western blot and reverse transcription-quantitative PCR (RT-qPCR) analyses. The antagomir-503-5p and antagomir negative control were synthesized and purchased from RiboBio (Guangzhou RiboBio Co., Ltd.) (Xie et al., 2019).

#### 2.2.4 RT-qPCR

The expression levels of the OC biomarker genes (*Acp5*, *Mmp9*, *Tnfrsf11a,* and *Nfatc1*) and *Ers1* and *Ers2* were detected by RT-qPCR; the experimental grouping and cell treatment were the same as in “2.2.2.” Total RNA was extracted from RANKL-induced RAW264.7 cells with 0.5 mL Trizol^®^ reagent (Cat:15596026CN, Invitrogen; United States) according to the manufacturer’s instructions. The RNA concentration and quality were measured by nucleic acid protein analyzer (BioSpec nano, SHIMADZU, JAPAN). Reverse transcription was performed with 0.5 μg total RNA and cDNA was synthesized by the reverse transcription system kit (Cat: K1622, Thermo Fisher Scientific, United States) according to the manufacturer’s instructions. The RT-qPCR reaction volume was 10 μL and it was performed following the instructions from the manufacturer’s kit (Cat: MQ101-01, Vazyme, Nanjing, China). The expression analysis was performed calculated using the 2^−ΔΔCT^, according to previous research (Ding et al., 2022). The primer sequences of *Acp5*, *Mmp9*, *Tnfrsf11a*, *Ers1*, *Ers2*, *Gapdh* and *Nfatc1* are described in Table 1. The primer sequences for miR-503-5p and U6 have been previously reported (Huang et al., 2020). U6 was the reference gene for miR-503-5p and *Gapdh* was the reference gene for *Ers1*, *Ers2*, *Trap*, *Mmp-9*, *Tnfrsf11a* and *Nfatc1*.

**TABLE 1:** Sequences of primers for genes.

Gene name	Forward	Reverse	ProdSize
*Gapdh*	CTC​ATG​ACC​ACA​GTC​CAT​GC	TTC​AGC​TCT​GGG​ATG​ACC​TT	155
*Tnfrsf11a*	CTG​AAA​AGC​ACC​TGA​CAA​AAG​A	CTG​TGT​AGC​CAT​CTG​TTG​AGT​T	84
*Mmp9*	CAA​AGA​CCT​GAA​AAC​CTC​CAA​C	GAC​TGC​TTC​TCT​CCC​ATC​ATC	105
*Trap*	GAC​CAC​CTT​GGC​AAT​GTC​TCT​G	GGC​TGA​GGA​AGT​CTC​TGA​GTT​G	262
*Nfatc1*	GAG​AAT​CGA​GAT​CAC​CTC​CTA​C	TTG​CAG​CTA​GGA​AGT​ACG​TCT​T	93
*Esr2*	GAT​TGT​TTA​TGC​TGA​GGG​AAG​C	TCT​GCT​TCT​ACT​TAC​AGA​CAC​G	83
*Esr1*	CTA​CTA​CCT​GGA​GAA​CGA​GC	GCG​TCG​ATT​GTC​AGA​ATT​AGA​C	88

#### 2.2.5 Western blot analysis

The protein expression levels of MMP9, RANK, ESR1 and ESR2 were assessed by western blot analysis. The experimental grouping and cell treatment were the same as in the section osteoclastogenesis and TRAP staining. Briefly, RAW264.7 cells were treated with ICT containing 50 ng/mL RANKL with or without MPP or antagomiR-503-5p for 5 days. Subsequently, the total protein was extracted with RIPA reagent (G2002-100ML, Servicebio Technology Co., Ltd. China). The protein concentration was determined by the Bradford assay (Solarbio, China) according to the manufacturer’s instructions. The same concentration of protein was denatured at 100 °C for 5 min, and 30 μg protein was loaded onto 10% SDS-PAGE gel. Electrophoresis was performed for 60 min at 120 V. The protein was transferred to a polyvinylidene fluoride membrane (IPVH85R, Millipore, United States). Then, the membrane was blocked with 5% BSA for 1 h and subsequently incubated with anti-β-actin antibody (Cat: AC026, ABclonal Biotech Co, Wuhan, China), anti-RANK antibody (Cat: 52985, Cell Signaling Technology, United States), anti-MMP9 antibody (Cat: AF5228, Affinity Biosciences, China), anti-ESR1 antibody and anti-ESR2 antibody (Cat: 21244-1-AP,ProteinTech, Wuhan, China) overnight at 4 °C respectively. The recovery of the primary antibody was performed and PVDF membranes were washed 3 times (10 min/times) with TBST. The secondary antibodies (Cat: A0208, Beyotime, Beijing, China) were added to the membranes for 1.5 h at room temperature. The PVDF membranes were processed with enhanced ECL chemiluminescence kit (G3308, GBCBIO Technologies Inc., guangzhou, China) using the chemiluminescence apparatus (5200CE, Tanon, Beijing, china).

#### 2.2.6 Cellular thermal shift assay (CETSA)-western blotting

RAW264.7 cells were grown to 80%–90% in the 25 cm^2^ flask under the aforementioned conditions followed by ICT or dimethylsulfoxide (DMSO, ST038, Beyotime Institute of Biotechnology, Beijing, China) treatment for 3 h. Subsequently, the cells were harvested and washed with PBS for 3 times and incubated with RIPA lysis buffer at each temperature point from 42 °C to 67 °C (42, 47, 52, 57, 62 °C, and 67 °C) for 5 min, and centrifuged at 12,000 rpm at 4 °C for 10 min and the supernatant was removed and mixed with 5 × loading buffer (p1040, Solarbio, and China). The resulting sample was separated on SDS-PAGE gel for the immunoblotting assay as our previously reported (Xie et al., 2025).

#### 2.2.7 Drug affinity responsive target stability (DARTs)-western blotting

Cell lysates were obtained from RAW264.7 cells by ice-cold M-PER lysis buffer (78503, Thermo Fisher Scientific Inc., United States) supplemented with PMSF (1 mM, P0100, Solarbio, Beijing, China) and protein phosphatase inhibitors (1 mM, P1260, Solarbio, Beijing, China). After collecting the cells with a scraper, the cells were incubated for 15 min at 4 °C. The cell lysates were centrifuged at 12,000 rpm for 15 min and the supernatant was diluted with M-PER lysis buffer to a final protein concentration of 5.8 mg/mL. The protein lysates were mixed with 10× TNC buffer. The lysates in 1× TNC buffer were split into 1.5 mL tubes and incubated with DMSO or ICT (100 μM) for 1 h at room temperature. Drug concentrations for DARTs were selected based on a previous study (Lomenick et al., 2009). Following incubation, each sample was split into 60 μL of aliquots (60 μg in proteins) and proteolyzed in various concentrations of pronase (Cat: 10165921001, Sigma, United States) for 10 min at room temperature. All samples were used for western blot analysis.

#### 2.2.8 Plasmid transfection

pcDNA-ESR1-WT, pcDNA-ESR1-Mu and pcDNA-vectors were designed and synthesized by Youbao Bio (Changsha, China). The pcDNA-ESR1-WT (1 µg), pcDNA-ESR1-Mu (1 µg) and pcDNA-vectors (1 µg) were transfected into RAW264.7 cells by ExFect transfection reagent (T101, Vazyme, Nanjing, China). According to the instructions of the kit and our previous research (Xie et al., 2025). Briefly, the transfection reagent was diluted with serum-free DMEM, the ratio of transfection reagent to plasmid was (5:1), incubated at room temperature for 20 min, and transfected for 8 h, change the medium to a complete medium containing serum (10% FBS). After transfecting the cells for 24 h, they were utilized for subsequent drug administration treatments, and total protein was extracted from RIPA lysate for subsequent experiment.

### 2.3 Molecular docking analysis

The crystal structure of the human ESR1 (PDB:1ERE) and ESR2 (PDB: 5TOA) was obtained from the PDB protein data bank. Autodock vina was used for molecular docking, docking methods were performed as our previously reported (Wang H. et al., 2023). Briefly, the protein was prepared by removing water, adding hydrogen, and other treatments. Finally, the center coordinates and box were selected to dock the ligand, and the best position of ICT was selected by docking score.

### 2.4 Statistical analysis

The data are presented as mean ± standard deviation (n = 3–7). Statistical analyses were performed using SPSS 20.0 (IBM or SPSS, Inc.). The data were first tested for normal distribution and homogeneity of variance, which conformed to normal distribution. The independent sample T-test was used for comparison of two groups of independent samples; the one-way ANOVA analysis was used for comparison between multiple groups. Bonferroni test was used for homogeneity of variance data, Dunnett^,^ s T3 test was used for uneven variance, and non-parametric Kruskal-Wallis test was used for non-normal distribution. *P* < 0.05 was considered to indicate a statistical significant difference.

## 3 Results

### 3.1 ICT inhibited ovariectomy-induced bone loss

Insufficient bone formation and excessive bone resorption caused by estrogen deficiency are the major factors resulting in the incidence of PMOP. Bilateral ovariectomy, which leads to a decrease in estrogen secretion, is a classic method for creating an PMOP model (Cui et al., 2024). Micro-CT was used to assess bone mass in mice with OVX-induced osteoporosis. As shown in Figure 1B, the OVX group, bone mass exhibited significantly reduced bone mass compared with the Sham groups. ICT treatment led to an increase in bone mass, with thicker and more abundant trabeculae. As shown in Figures 1C–F, BV/TV, Conn.D and Tb.Th were significant increased in a dose-dependent manner ICT treatment groups than OVX group (*P* < 0.001, *P* < 0.05, respectively). In particular, the high-dose treatment group had a significant anti-osteoporosis effect (*P* < 0.001). In addition, we measured bone mineral content. Interestingly, compared with the Sham group, bone mineral content was significantly decreased in the OVX group, and increased in a dose-dependent manner by all concentration of ICT treatment (Figures 1G,H). but SMI were significantly lower in the ICT-treated groups than in the OVX group (Figure 1I, *P* < 0.001, *P* < 0.05, respectively). More importantly, we detected the serum OPG, RANKL and TRAP levels by ELISA kits, and we found that ICT treatment significantly increased OPG levels and inhibited RANKL and TRAP levels (Figures 1J–L). The OPG/RANKL/RANK signaling pathway is generally considered to be a key pathway that regulates the functional balance of osteoclasts and osteoblasts (Kovacs et al., 2019; Vachliotis and Polyzos, 2023). Thus, our study found that ICT has a good pharmacological effect on osteoporosis, especially on postmenopausal osteoporosis, and the mechanism may be related to the regulation of osteoblast and osteoclast homeostasis.

### 3.2 ICT inhibits OC differentiation and upregulation of ESR expression

**FIGURE 2:**
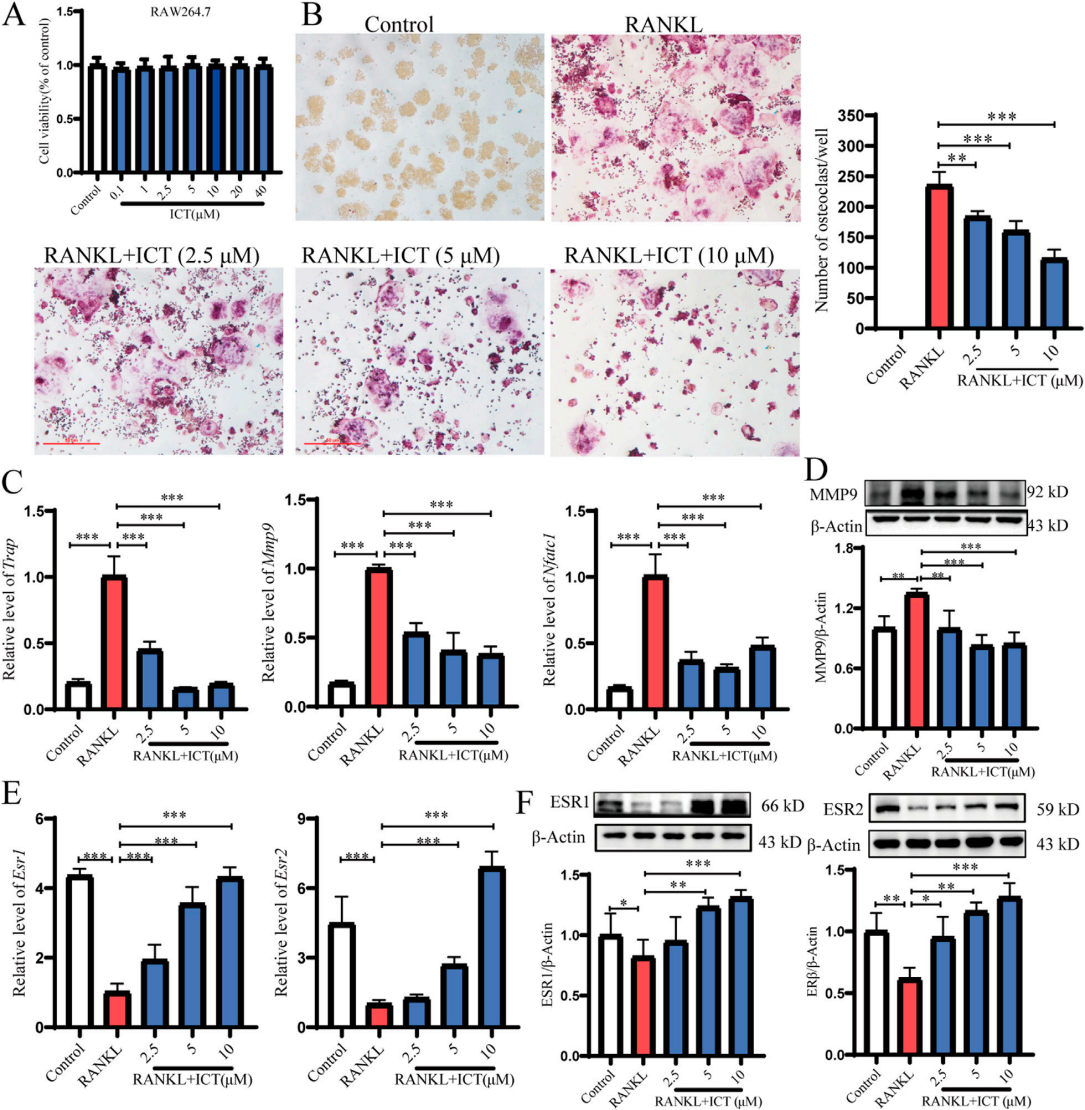
ICT significantly inhibited osteoclast differentiation and promoted the expression of ESR1 and ESR2. **(A)** ICT effect on the cytotoxicity of RAW 264.7 cells (n = 5). **(B)** Inhibition of OC differentiation by ICT was detected using TRAP staining (n = 5). **(C)** The effect of ICT on the expression of genes related to OC differentiation was assessed using RT-qPCR (n = 4). **(D)** MMP9 expression was detected following ICT treatment using western blot analysis (n = 4). **(E)** The effect of ICT on the expression levels of *Esr1* and *Esr2* was investigated using RTq-PCR (n = 4). **(F)** The effects of ICT on the protein levels of ESR1 and ESR2 were examined using western blot analysis (n = 4). *P < 0.05, **P < 0.01, ***P < 0.001 vs. the RANKL group, scale column:50 μm.

To detect the effect of ICT on osteoclast differentiation, we treated osteoclasts differentiated from RAW264.7 induced by RANKL with ICT. RAW264.7, acts as a precursor osteoclast cell. Firstly, we detect the effects of ICT on the activity and proliferation of RAW264.7 cells, and RAW264.7 cells were treated with various concentrations of ICT for 48 h to assess its toxicity on osteoclast precursors. As shown in Figure 2A, ICT concentrations of 0.1–40 μM (40, 20, 10, 5, 2.5, 1, and 0.1 μM) had no cytotoxicity on RAW264.7 cells, the concentrations of 10 μM, 5 μM, and 2.5 μM were used in subsequent experiments. Previous studies have demonstrated the beneficial effect of ICT on osteoporosis prevention and its significant inhibition of OC differentiation (Huang et al., 2018). About OC differentiation, compared with RANKL group, the Control group did not osteoclast differentiation and could not be stained wine red by the TRAP staining solution, but ICT inhibited RANKL-induced OC differentiation in a dose-dependent manner (Figure 2B). Moreover, the effects of ICT were examined on the expression levels of OC differentiation-related genes by western blot and RT-qPCR analyses. It is interesting to note that, compared with Control group, RANKL group significantly promoted the expression levels of *Trap*, *Mmp9* and *Nfatc1,* but compared with RANKL group, ICT dose-dependently inhibited the mRNA expression levels of *Trap*, *Mmp9* and *Nfatc1* (Figure 2C), as well as the protein expression of MMP9 (Figure 2D). Furthermore, the effect of ICT on RANKL-induced ESR1 and ESR2 expression was explored. Interestingly, compared with control group, the expression of RANKL significantly inhibits ESR1 and ESR2, compared with model group, but ICT dose-dependently upregulated the mRNA and protein expression levels of ESR1 and ESR2 (Figures 2E,F). This suggests that the inhibition of OC differentiation by ICT may be related to the ESR signaling pathway.

### 3.3 ESR1 is the direct targets of ICT

**FIGURE 3:**
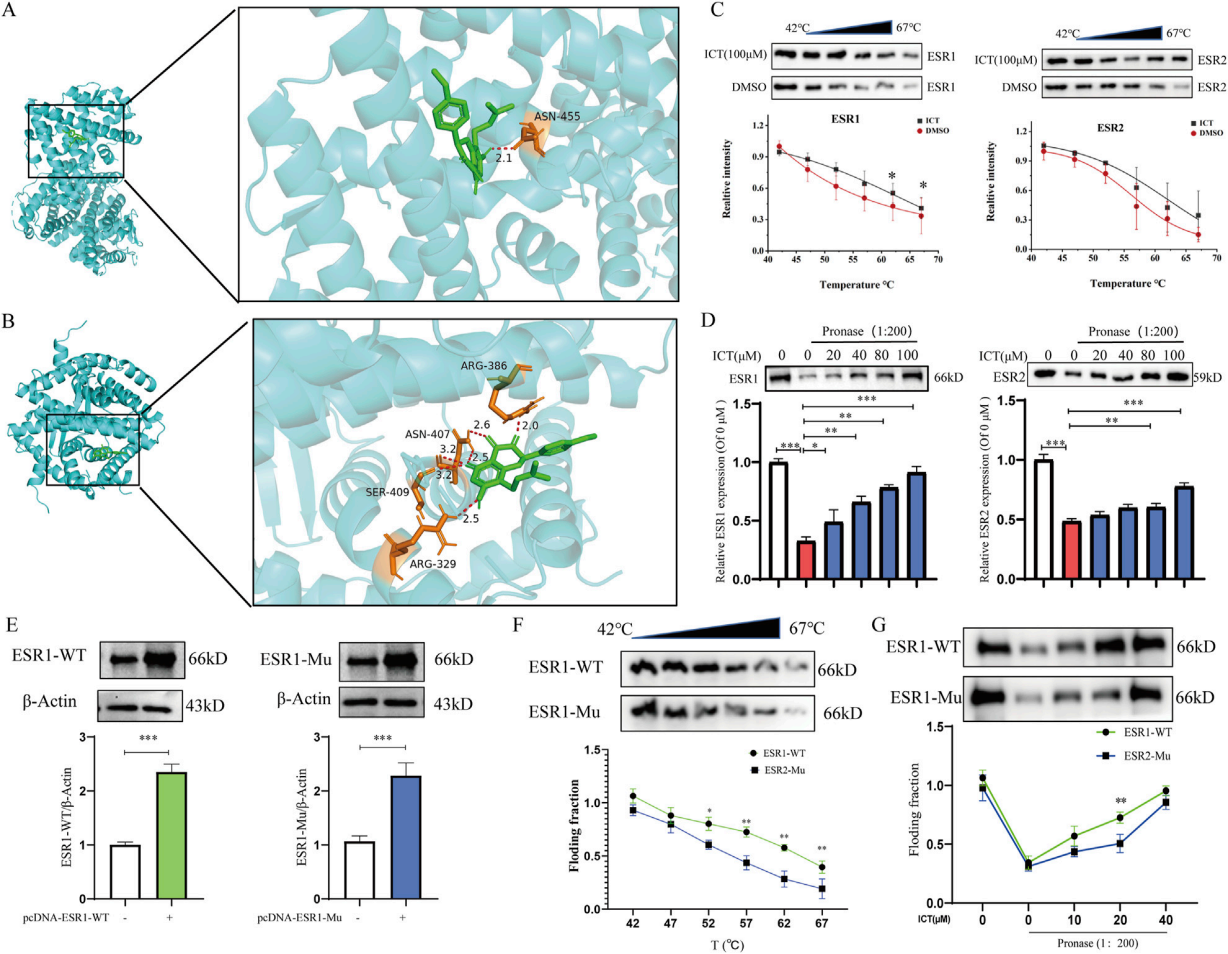
ESR1 is the direct targets of ICT. **(A)** Auto docking ICT and ESR1 (PDB:1ERE). **(B)** Auto docking ICT and ESR2 (5TOA). **(C)** The CETSA experiment evaluated the interaction of ICT with ESR1 and ESR2 (n = 3). **(D)** The DARTs experiment evaluated the interaction of ICT with ESR1 and ESR2 (n = 3). **(E)** pcDNA-ESR1-WT and pcDNA-ESR1-Mu were transfected into RAW264.7 cells (n = 3). **(F,G)** DARTs and CETSA experiments confirmed the interaction of ICT with ESR1-WT or ESR1-Mu (n = 3). *P < 0.05, **P < 0.01, ***P < 0.001 vs. the DMSO group or pcDNA-ESR1-Mu group.

Estrogen deficiency is the main cause of osteoporosis in postmenopausal women, and it is also the main mechanism of phytoestrogens against osteoporosis (Patra et al., 2023; Yao et al., 2025). Our previous research found that ICT significantly promoted the expression of ESR1 and ESR2 mRNA and protein levels (Figures 2E,F). To elucidate the direct target of ICT in inhibiting OC differentiation and reduce bone loss, molecular docking simulations were performed using auto docking software to examine the interaction between ICT and ESR1 and ESR2. For ESR1, the analysis indicated that both ICT and ESR1 interaction binding energy is −7.33 Kcal/mol (Figure 3A). However, ICT and ESR2 interaction binding energy is −6.93 Kcal/mol (Figure 3B), suggesting that ICT interacted well with both ESR1 and ESR2. Furthermore, the interaction of ICT with ESR1 and ESR2 was confirmed using CETSA and DARTs experiments. Compared with the DMSO group, the CETSA experiment performed in RAW264.7 cells indicated that the thermal stability of ESR1 and ESR2 was increased to varying degrees when ICT (100 μM) was added, ranging from 42 °C to 67 ˚C (Figure 3C). Moreover, similar results were observed in the DARTs assay, in which ICT dose-dependently attenuated the degradation of ESR1 and ESR2 proteins by pronase, specifically ESR1, the interaction between ICT and ESR1 demonstrated significantly greater efficacy than that between ICT and ESR2. Additionally, the enzymatic stability of ESR1 may be improved by administering lower doses of ICT (Figure 3D). Previous studies have found that ESR1 is a key target in regulating osteoclast differentiation and osteoporosis (Deng and Guo, 2020; Pelusi et al., 2022). According to the results of molecular docking, Asparagine at ESR1 455 was mutated into alanine (ESR1-Mu) and the plasmid was transfected into RAW264.7 cells (Figure 3E). Then, the interaction between ICT and ESR1-WT or ESR1-Mu was verified by CETSA and DARTs. Interestingly, ICT interacts significantly reduce with ESR1-Mu compared to wild-type ESR1 (ESR1-WT) (Figures 3F,G). It is suggested that ESR1, rather than ESR2, may serve as the primary target of ICT, and it has been proposed that ESR1, but not ESR2, plays a pivotal role in osteoclast differentiation and osteoporosis. Taken together, ESR1 is a direct target of ICT, and Asparagine at 455 is a key site for ICT-targeted ESR1 binding.

### 3.4 ICT inhibits OC differentiation by target ESR1

**FIGURE 4:**
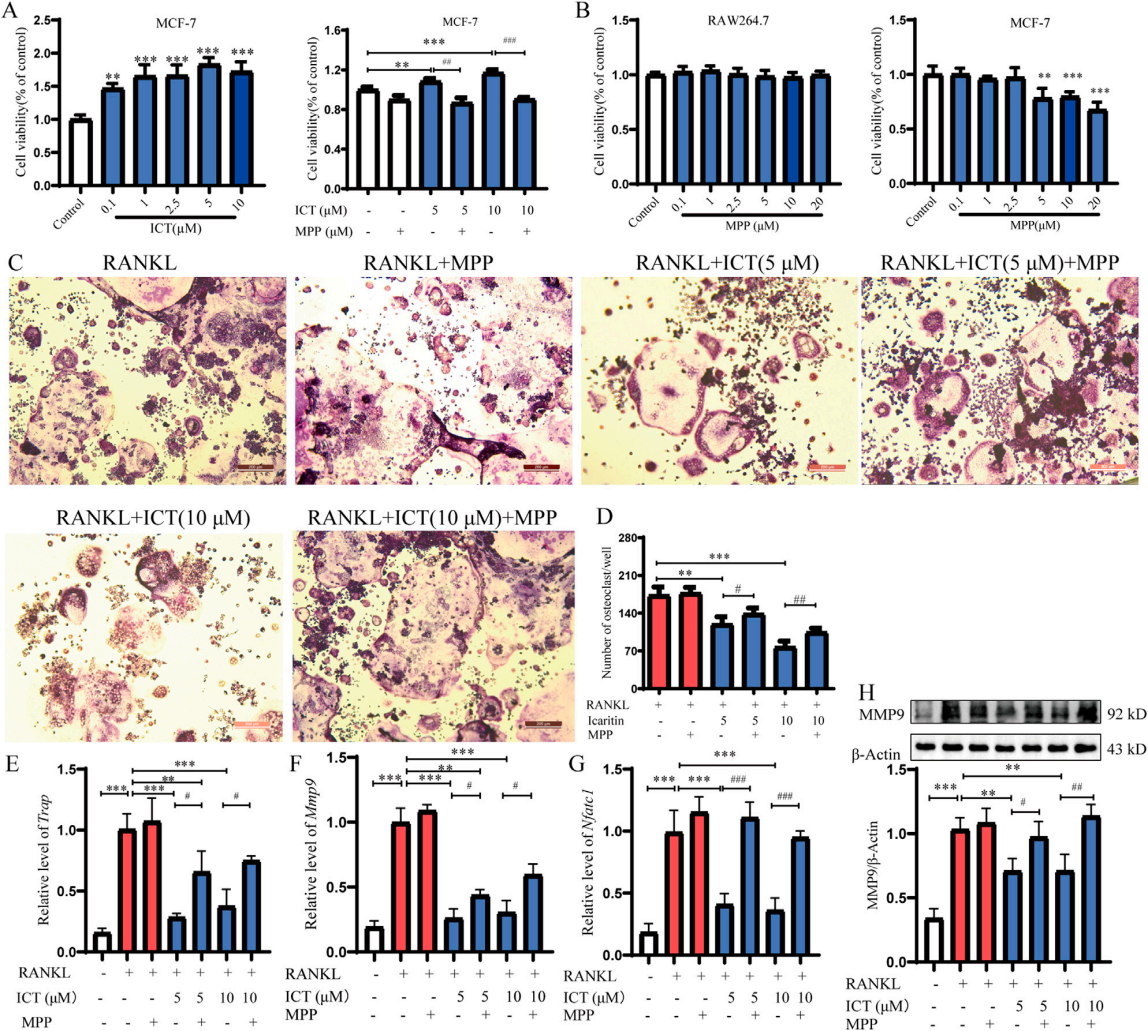
ICT inhibits OC differentiation by the ESR signaling pathway. **(A)** MPP blocks the proliferative effect of ICT on MCF-7 cells (n = 5). **(B)** The cytotoxicity of MPP on RAW264.7 and MCF-7 cells were detected by the CCK-8 assay (n = 5). **(C)** TRAP staining following ESR1 antagonism by MPP (n = 3). **(D)** TRAP-positive cells following antagonism of ESR1 by MPP. **(E–G)** Effect of ICT on the expression levels of genes related to OC differentiation following antagonism of ESR1 by MPP as detected by RT-qPCR (n = 3). **(H)** MMP9 expression was detected by western blot analysis following antagonism of ESR1 by MPP (n = 3). ^*^ *P* < 0.05, ^**^ *P* < 0.01, ^***^ *P* < 0.001 vs. the RANKL group, ^#^ *P* < 0.05, ^##^ *P* < 0.01, ^###^ *P* < 0.001 vs. the ICT (5 μM) and ICT (10 μM) group, scale column:50 μm.

The ESR pathway plays an important role in OC differentiation and osteoporosis and it is the direct target of phytoestrogens (Khalid and Krum, 2016). To further confirm the estrogenomimetic effect of ICT, MCF-7 cells were treated with ICT; the data indicated that ICT (0.1, 1, 2.5, and 5 µM) promoted the proliferation of MCF-7 cells and the pro-proliferative effect of ICT on MCF-7 was inhibited by MPP, which is a competitive inhibitor of ESR1. Compared with control group, MPP at the experimental dose was not cytotoxic to RAW264.7 and MCF-7 cells (Figures 4A,B), suggesting that ICT may be a phytoestrogen and ESR1 its direct target. To confirm that ICT inhibition of OC differentiation is related to the target of ESR1, MPP was used to inhibit the ESR1 functions. The results indicated that, compared with RANKL group, ICT (5 or 10 μM) significantly inhibited OC differentiation, but compared with ICT (5 μM or 10 μM) groups, MPP reversed the effects of ICT on OC differentiation (Figures 4C,D). Moreover, the results of RT-qPCR and western blot analyses indicated that MPP could significantly reduce the inhibitory effect of ICT on the expression levels of the OC biomarkers (*Mmp9*, *Trap*, *Nfatc1*) in mRNA and protein levels (Figures 4E–H). Therefore, our research has found that ICT may target ESR1 to inhibit the differentiation of osteoclasts.

### 3.5 ICT inhibit OC differentiation by miR503/RANK pathway via targeting ESR1

**FIGURE 5:**
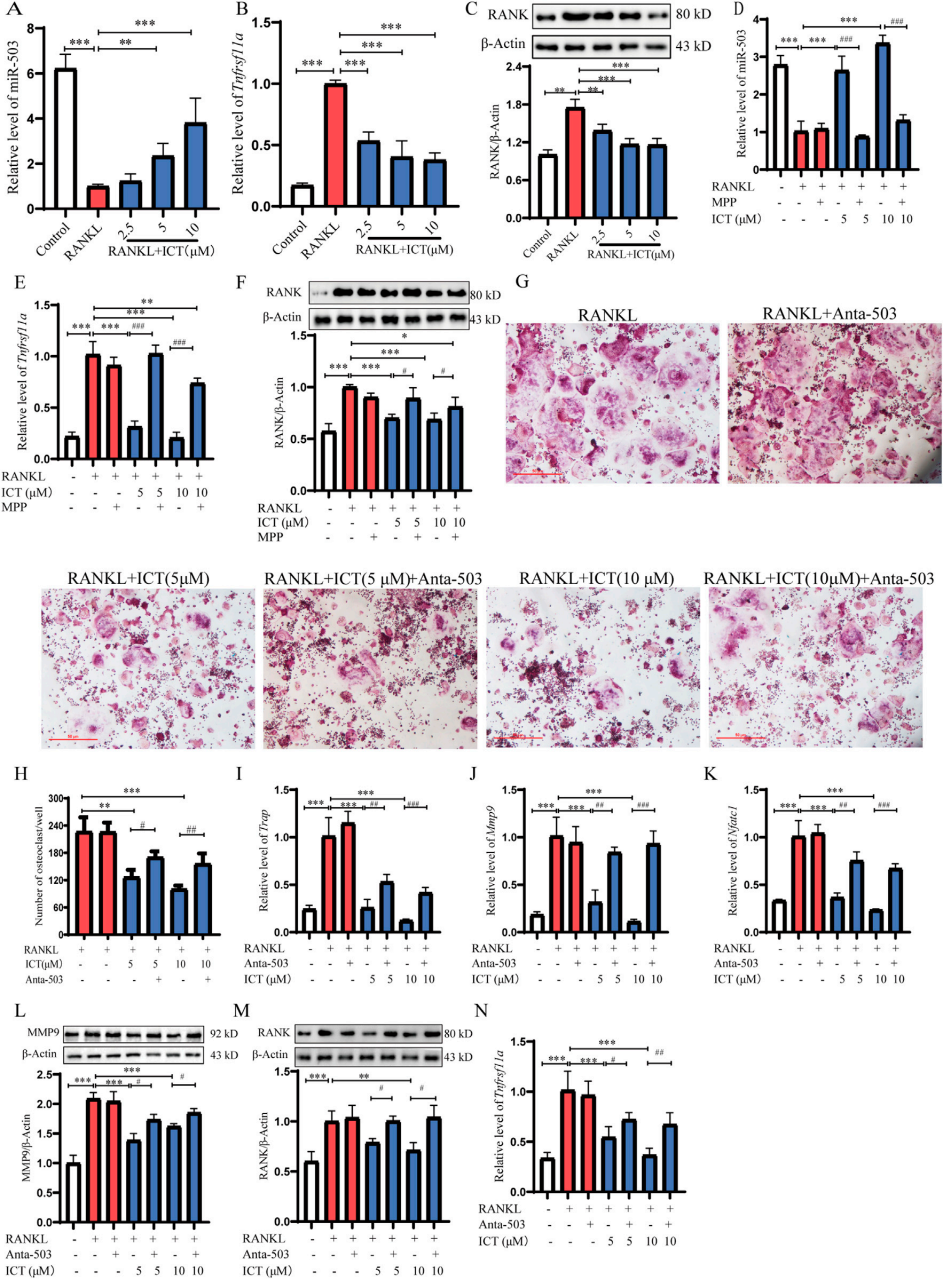
ICT inhibits OC differentiation via the miR-503-5p/RANK signaling pathway. **(A)** ICT upregulates miR-503 expression in OC differentiation (n = 3). **(B,C)** ICT inhibits RANK mRNA and protein expression in OC (n = 3). **(D)** MPP blocks ICT on miR-503-5p expression (n = 3). **(E,F)** MPP antagonizes the effect of ICT on the mRNA and protein expressions of RANK. **(G,H)** Antagomir-503-5p antagonizes the effect of ICT on OC differentiation (n = 3). **(I–K)** Effect of ICT on the expression levels of genes related to OC differentiation following inhibition of miR-503-5p by antagomiR-503-5p as demonstrated by RT-qPCR (n = 3). **(L,M)** The expression levels of MMP9 and RANK were detected by western blot analysis following inhibition of miR-503-5p by antagomir-503-5p (n = 3). **(N)** RANK expression was detected by RT-qPCR (n = 3). ^*^ *P* < 0.05, ^**^ *P* < 0.01, ^***^ *P* < 0.001 vs. the model group; ^#^ *P* < 0.05, ^##^ *P* < 0.01 vs. ICT + antagomir-503 group, scale column:50 μm.

ESR1 is a transcription factor that can regulate the expression levels of miRNA, such as miR-503 (Chen et al., 2014; Perez-Cremades et al., 2018). RANK, as a direct target of miR-503 action, is also a receptor for the OC differentiation inducer RANKL. Previous studies have shown that the miR-503/RANK axis plays an important role in OC differentiation (Chen et al., 2014; Huang et al., 2020). To elucidate the mechanism by which ICT inhibits OC differentiation by regulating the miR-503/RANK pathway via ESR1, the effects of ICT were examined on the expression levels of miR-503 and RANK. Compared with control group, RANKL induced significant inhibition of miR503 expression and promoted RANK mRNA and protein expression, compared with RANKL group, ICT significantly promoted the expression of miR-503 and inhibited the expression of RANK in dose-dependent (Figures 5A–C). Then, we used MPP to competitively antagonize ESR1 and detect ICT regulation of miR-503 and RANK expression, compared with ICT + MPP group, it is interesting to note that MPP can antagonize the effect of ICT (5 μM and 10 μM) on miR-503 and RANK expression (Figures 5D–F). It is suggested that ICT inhibition of OC differentiation may be closely related to ESR1 regulation of the miR-503/RANK axis. To further elucidate the mechanism by which ICT inhibits OC differentiation via the miR-503-5p/RANK signaling pathway, antagomir-503-5p was used to antagonize the function of miR-503. It is interesting to note that ICT dose-dependent inhibition of osteoclast differentiation, but the inhibitory effect of ICT on OC differentiation was weakened, after Inhibiting the biological function of miR-503 (Figures 5G,H), as well as the expression of OC biomarkers (*Trap*, *Mmp9*, *Nfatc1*) and RANK (Figures 5I–N). These findings suggest that ICT inhibits OC differentiation via the miR-503-5p/RANK axis. Taken together, our research has revealed that the ICT targeting ESR1 promotes the expression of miR-503, inhibits the expression of RANK and the differentiation of osteoclasts.

### 3.6 ICT inhibited ovariectomy-induced bone loss by targeting ESR1

**FIGURE 6:**
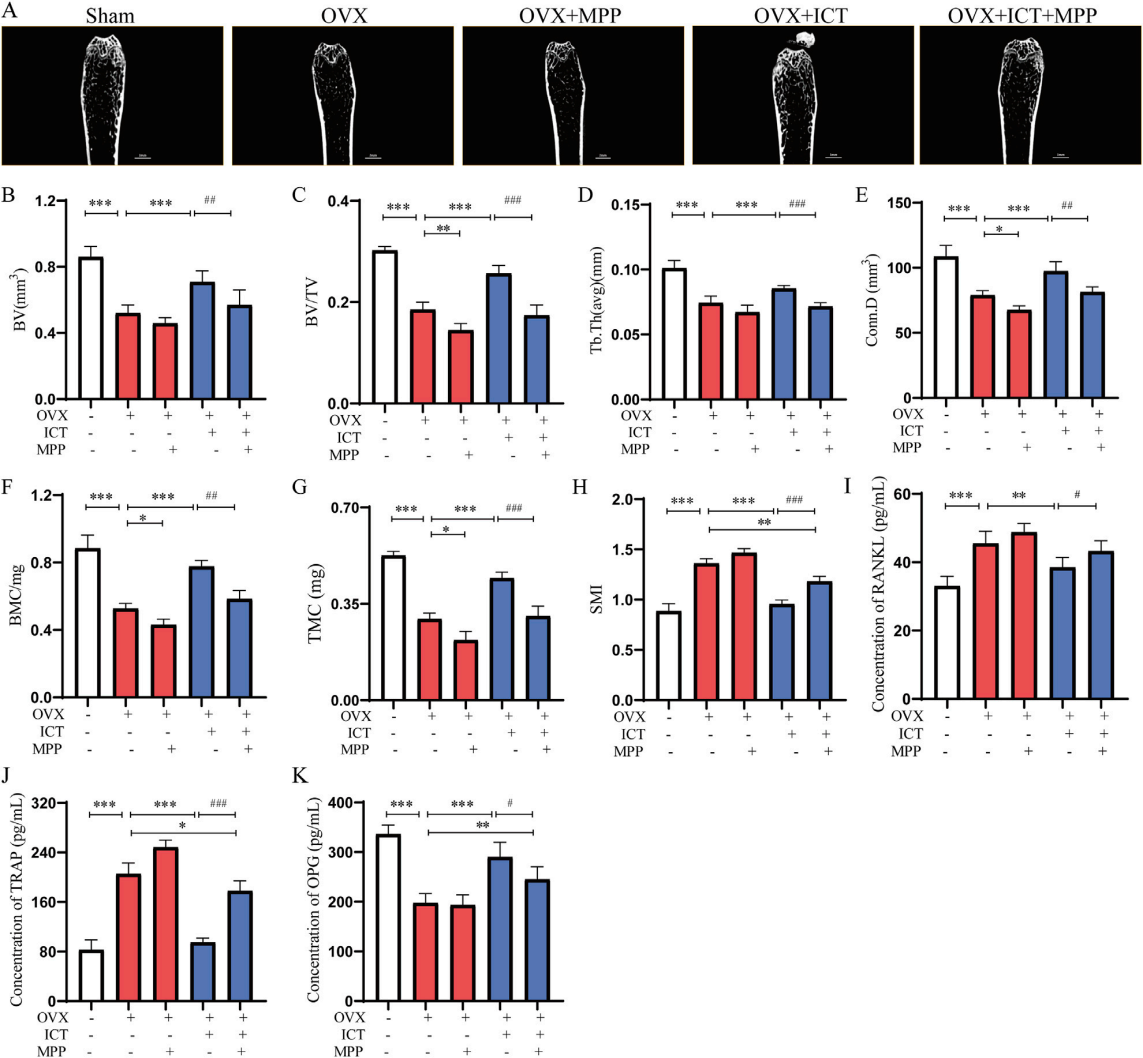
Stereological parameter of trabecular bone in the mouse femur after ICT and MPP co-treatment analyzed by micro-CT. **(A)** Region of interest (ROI) and longitudinal section (n = 6), **(B)** Bone volume (BV) (n = 6), **(C)** Bone volume/total volume (BV/TV) (n = 6), **(D)** Trabecular thickness (Tb.Th) (n = 6), **(E)** Trabecular bone connection density (Conn.D) (n = 6), **(F)** Bone mineral content (BMC) (n = 6), **(G)** Tissue mineral content (TMC) (n = 6), **(H)** Structure model index (SMI) (n = 6), **(I–K)** The level of TRAP, RANKL and OPG in serum after ICT and MPP co-treatment (n = 6), *P < 0.05, **P < 0.01, ***P < 0.001 vs. OVX group, ^#^ *P* < 0.05, ^##^ *P* < 0.01, ^###^ *P* < 0.001 vs. the ICT (30 mg/kg/d) group, scale column:5 mm. OVX, ovariectomize; ICT, icaritin; MPP, MPP dihydrochloride. TRAP, tartrate-resistant acid phosphatase; OPG, osteoporogeterin; MMP9, Matrix metalloproteinase 9.

As previous studies have shown that ESR1 is a key target in the regulation of osteoclast differentiation and osteoporosis (Cheng et al., 2022; Wang L. H. et al., 2023). Our study found that ICT significantly promotes ESR1 expression and that ICT interacts well with ESR1 (Figure 3D). We used MPP, a competitive antagonist of ESR1, to block the biological function of ESR1. The results of animal experiments showed that, compared with sham group, bilateral ovariectomy significantly reduces the volume of bone trabeculae, bone mineral content, and alters the bone microstructure, compared with OVX-ICT group, blocking ESR1 significantly inhibited the effect of ICT on OVX-induced osteoporosis (Figure 6A). Compared with the OVX-ICT group, MPP treatment significantly reduced the regulatory effect of ICT on BV, BV/TV and Tb.Th (Figures 6B–E), as well as bone mineral content and TMC (Figures 6F–H). About SMI, the opposite result was shown, compared with the OVX-ICT group, MPP treatment significantly reduced the regulatory effect of ICT on SMI (Figure 6H). More importantly, we found that blockade of ESR1 significantly attenuated the inhibitory effect of ICT on RANKL and TRAP expression, as well as the promoting effect on OPG expression as measured by ELISA (Figures 6I–K). Therefore, ESR1 is a direct target of the inhibitory effect of ICT on osteoclast differentiation and anti-osteoporosis.

## 4 Discussion

Excessive activation of osteoclasts leads to bone loss in the majority of skeletal diseases, notably bone metabolic diseases. Therefore, the inhibition of OC differentiation or function is a promising strategy for the treatment of OP (Agidigbi and Kim, 2019; Yao et al., 2021; Zeng et al., 2016). In our study, we first confirmed by *in vivo* experiments that ICT significantly inhibited OVX-induced osteoporotic lesions and reduced bone loss (Figure 1). RAW264.7 cells are OC precursors and can differentiate into OC in response to RANKL (Zeng et al., 2016). In our research, we found that RANKL-induced RAW264.7 cell differentiation into OC was inhibited by ICT (Figure 2B) without dose-dependent cytotoxic effects. Concomitantly, ICT (10, 5, and 2.5 μM respectively) significantly inhibited the mRNA and protein expression levels of OC differentiation biomarkers (*Trap*, *Mmp9*, *Tnfrsf11a* and *Nfatc1*). It is important to know that compared with the control group, the expression levels of *Ers1* and *Ers2* in the RANKL group were significantly decreased; however, ICT dose-dependently promoted the mRNA and protein expression levels of *Ers1* and *Ers2* (Figures 2E,F).

Inadequate estrogen secretion is the main cause of OP in postmenopausal women (Cui et al., 2024). Estrogen replacement therapy is also the main method for the clinical treatment of PMOP (Guo et al., 2021). ESR is a direct target of estrogens and phytoestrogens and plays an important role in osteoclastogenesis (Cheng et al., 2022). Moreover, deficiency in ESR1 expression enhances OC differentiation and activity (Norton et al., 2022). ESR can also inhibit the action of RANKL, thereby inhibiting OC formation and bone resorption activity. ESR is also a key target of phytoestrogens and functions to inhibit OC differentiation (Abu-Amer, 2013). Tussilagone increases ESR1 and Fas ligand expression to promote OC apoptosis and prevent estrogen deficiency-induced OP in mice (Ryoo et al., 2020). More and more research confirmed that ESR1 is the direct target of ICT by bibliometrics and bioinformatic analysis (Huong and Son, 2023; Zhou et al., 2024). However, most reports have been based on bioinformatics and literatures analysis, rather than experimental studies, and the ICT-ESR1 complex binding check point is unclear. In the present study, molecular docking techniques were used to confirm that ICT exhibited an optimal interaction with ESR1 and ESR2 (Figures 3A,B). Subsequently, CETSA and DARTs experiments were used to confirm that ESR1 and ESR2 were direct targets of ICT. Interestingly, ICT significantly enhanced the thermostability of ESR1, and inhibited the enzymatic hydrolysis of ESR1 by pronase in a dose-dependent manner, rather than ESR2 (Figures 3C,D), and we further confirmed that Asparagine at 455 is the direct binding site of ICT and ESR1 through the mutant plasmid, CETSA and DARTs (Figures 3E,F). CETSA and DARTs experiments are classic non-modified methods for small molecule target confirmation; the target protein becomes more stable as it binds to the drug molecule (Lomenick et al., 2009; Prabhu et al., 2020). Furthermore, ICT promotes the proliferation of MCF-7 cells; this effect can be blocked by the ESR1 antagonist MPP (Figure 4A), suggesting that ICT may be a phytoestrogen and exert estrogenic effects. MCF-7 cells are ESR positive cells and estrogen or phytoestrogens can promote their proliferation, it is commonly used in the screening of estrogen-mimicking drugs (Ren et al., 2018; Zafar et al., 2017). To confirm that ICT inhibits OC differentiation targeting ESR1, OC were treated with the ESR1 antagonist MPP (Dustin et al., 2019). It is interesting to note that MPP could significantly reduce the effect of ICT on OC differentiation and reverse the mRNA and protein expression levels of the biomarkers of OC differentiation (Figures 4C–H). Therefore, the ESR1 may be a key target for ICT to inhibit OC differentiation. More importantly. Emerging literature suggests that ESR1 is the most important receptor for the anti-osteoporotic effect of estrogen, the injection of estrogen does not exhibit bone protection in ESR2-knockout (KO) mice; however, estrogen decreases bone loss in ESR2-KO ovariectomized mice, which suggests that the direct target for the anti-osteoporotic effect of estrogen is ESR1, rather than ESR2 (Garcia et al., 2013; Nakamura et al., 2007).

**FIGURE 7:**
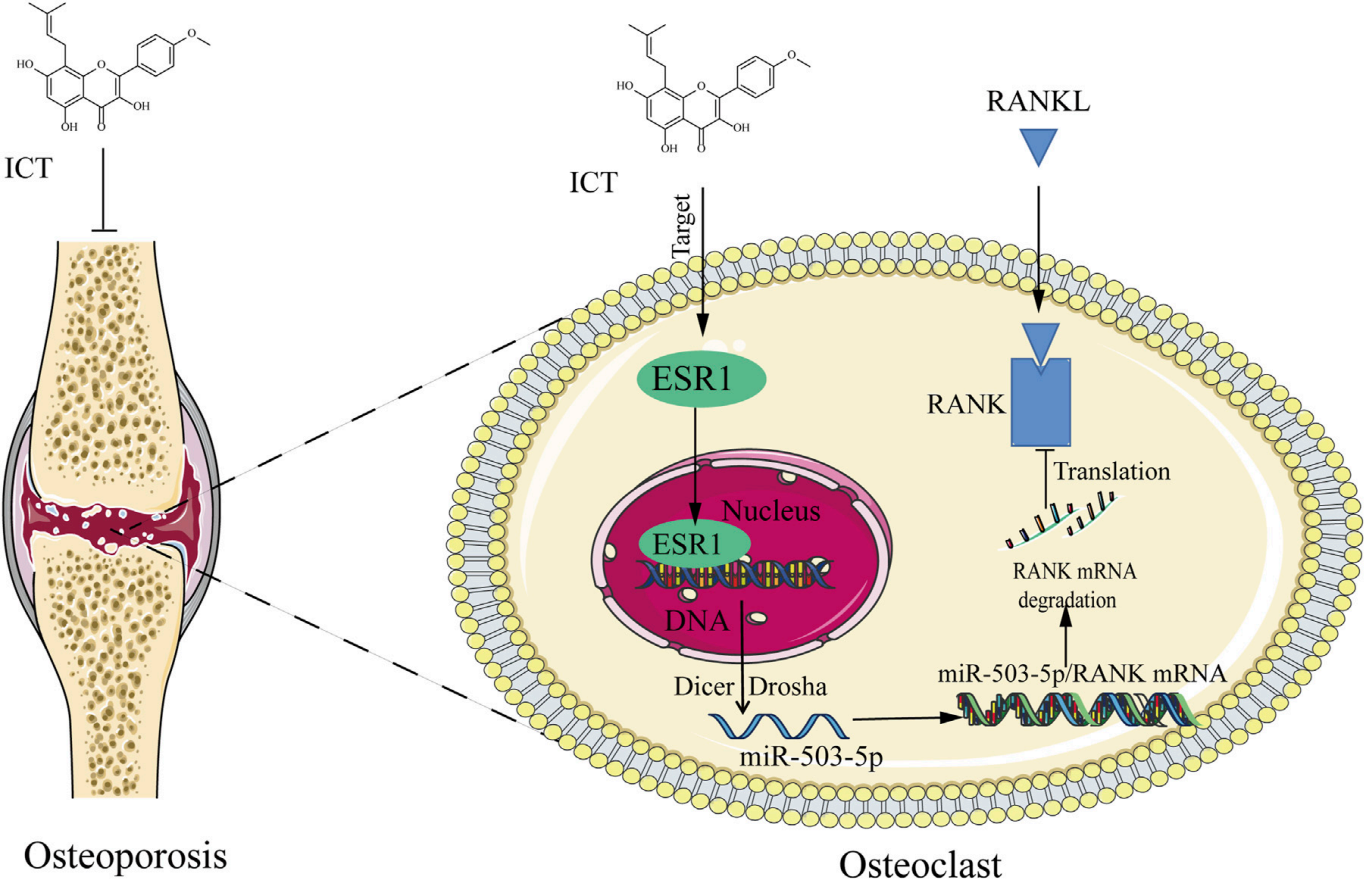
Icaritin suppresses osteoclast differentiation and anti-osteoporosis by targeting the estrogen receptor to modulate the miR-503-5p/RANK axis. ICT: Icaritin; ESR1: estrogen receptor α.

miR-503-5p is a small non-coding RNA, which has different functions in OP and bone metabolism disease (Liu et al., 2017; Wei et al., 2022). Overexpression of miR-503 inhibits RANKL-induced osteoclastogenesis by directly targeting RANK in cluster of differentiation 14^+^ peripheral blood mononuclear cells. 17-β-estradiol treatment caused a significant increase in miR-503 expression in RANKL-induced osteoclastogenesis, suggesting that miR-503 is a negative regulator of OC differentiation and that it may be related to the ESR signaling pathway (Chen et al., 2014). More importantly, Our previous research also found that ESR1 is an upstream regulatory factor of miR-503, and RANK is the direct target of miR-503, suggesting that the ESR1/miR-503/RANK pathway is the key mechanism regulating the differentiation of osteoclasts (Xie et al., 2019; Huang et al., 2020). In this study, we found that ICT significantly increased the expression level of miR-503 in OC and inhibited the mRNA and protein expression levels of RANK; and MPP can block the influence of ICT on miR-503 expression (Figures 5A–D). These results suggest that the expression level of miR-503 is regulated by ESR1. In contrast, the influence of ICT on the expression level of RANK, the direct target of miR-503, was opposite to the expression trend of miR-503 (Figures 5E,F). Furthermore, antagomiR-503-5p was used to antagonize the function of miR-503; The results showed that ICT inhibited OC differentiation, the mRNA and protein expression levels of OC differentiation related genes were decreased, and the mRNA expression levels of RANK was increased (Figures 5G–M). Therefore, ICT inhibit OC differentiation through the miR-503-5p/RANK axis by targeting ESR1. However, the mechanism of ICT regulation of miR-503-5p expression remains to be further studied, which may be related to Dicer and Drosha enzymes, which are key regulatory enzymes of miRNA maturation (Pong and Gullerova, 2018). More importantly, we blocked the biological functions of ESR1 with MPP, and the results showed that the inhibitory effects of ICT on osteoclast differentiation and anti-osteoporosis were significantly attenuated, especially in trabecular BV and BMD (Figures 6A–H), while the levels of TRAP and RANKL were significantly increased, and the level of OPG was significantly decreased (Figures 6I–K). Taken together, our research found that ESR1 is the direct target of ICT, and Asparagine at 455 is the direct binding site of ICT. ICT inhibit osteoclast differentiation and reduce bone loss by targeting ESR1 to upregulate miR503 level and weaken miR503/RANK pathway (Figure 7). However, our study still has some limitations: (1) Further analysis of the crystal structure of ICT-ESR1 and molecular dynamics simulation of the binding mechanism of the ESR1-ICT complex are conducive to drug development targeting ESR1, and the application of ICT for the clinical treatment of OP; (2) What is the specific mechanism by which ESR1 regulates the expression of miR-503? We will explore the downstream targets and mechanisms regulated by ESR1 and miR-503-5p through combined multiomics analysis techniques and immunofluorescence staining.

## Data Availability

The datasets presented in this study can be found in online repositories. The names of the repository/repositories and accession number(s) can be found in the article/supplementary material.
